# The VALID‐CRT risk score reliably predicts response and outcome of cardiac resynchronization therapy in a real‐world population

**DOI:** 10.1002/clc.23229

**Published:** 2019-07-13

**Authors:** Emanuele Bertaglia, Giuseppe Arena, Domenico Pecora, Albino Reggiani, Antonio D'Onofrio, Pietro Palmisano, Antonio De Simone, Salvatore I. Caico, Massimiliano Marini, Giampiero Maglia, Anna Ferraro, Francesco Solimene, Antonella Cecchetto, Maurizio Malacrida, Giovanni L. Botto, Maurizio Lunati, Giuseppe Stabile

**Affiliations:** ^1^ Department of Cardiac, Thoracic, and Vascular Sciences University of Padova Padua Italy; ^2^ Department of Cardiology Apuane Hospital Massa Italy; ^3^ Fondazione Poliambulanza Brescia Italy; ^4^ Pieve di Coriano Hospital Pieve di Coriano (MN) Italy; ^5^ Department of Cardiology Monaldi Hospital Naples Italy; ^6^ Cardiology Unit 'Card. G. Panico' Hospital Tricase (LE) Italy; ^7^ San Michele Clinic Maddaloni (CE) Italy; ^8^ Sant'Antonio Abate Hospital Gallarate (VA) Italy; ^9^ Department of Cardiology Santa Chiara Hospital Trento Italy; ^10^ Pugliese‐Ciaccio Hospital Catanzaro Italy; ^11^ Degli Infermi Hospital Rivoli (TO) Italy; ^12^ Montevergine Clinic Mercogliano (AV) Italy; ^13^ San Bortolo Hospital Vicenza Italy; ^14^ Boston Scientific Milan Italy; ^15^ U.O. Electrophysiology ASST Rhodense Rho‐Garbagnate Milanese (MI) Italy; ^16^ Cardiotoracovascular Department ASST Grande Ospedale Metropolitano Niguarda Milan Italy; ^17^ Mediterranean Clinic Naples Italy

**Keywords:** cardiac resynchronization therapy, clinical response, long‐term outcome, risk‐score

## Abstract

**Objectives:**

The aim of the study was to confirm the value of the VALID‐cardiac resynchronization therapy (CRT) risk score in predicting outcome and to assess its association with clinical response (CR) in an unselected real‐world CRT population.

**Methods and Results:**

The present analysis comprised all consecutive CRT patients (pts) enrolled in the CRT‐MORE registry from 2011 to 2013. Pts were stratified into five groups (quintiles 1‐5) according to the VALID‐CRT risk predictor index applied to the CRT‐MORE population. In the analysis of clinical outcome, adverse events comprised death from any cause and non‐fatal heart failure (HF) events requiring hospitalization. CR at 12‐month follow‐up was also assessed. We enrolled 905 pts. During a median follow‐up of 1005 [627‐1361] days, 134 patients died, and 79 had at least one HF hospitalization. At 12 months, 69% of pts displayed an improvement in their CR. The mean VALID‐CRT risk score derived from the CRT‐MOdular Registry (MORE) population was 0.317, ranging from −0.419 in Q1 to 2.59 in Q5. The risk‐stratification algorithm was able to predict total mortality after CRT (survival ranging from 93%‐Q1 to 77%‐Q5; hazards ratio [HR] = 1.42, 95% confidence interval [CI]: 1.25‐1.61, *P* < .0001), and HF hospitalization (ranging from 95% to 90%; HR = 1.24, 95% CI: 1.06‐1.45, *P* = .009). CR was significantly lower in pts with a high‐to‐very high risk profile (Q4‐5) than in pts with a low‐to‐intermediate risk profile (Q1‐2‐3) (55% vs 79%, *P* < .0001).

**Conclusion:**

The VALID‐CRT risk‐stratification algorithm reliably predicts outcome and CRT response after CRT in an unselected, real‐world population.

## 1 INTRODUCTION

Cardiac resynchronization therapy (CRT) is a standard therapy for patients with systolic heart failure (HF) and prolonged QRS duration^1^, ^2^, ^3^ The recognition that outcome is variable has prompted efforts to risk‐stratify patients on the basis of pre‐implantation assessments. The field of CRT demands a simple risk‐stratification algorithm based on a few routinely available variables. Mortality in CRT has been associated with several pre‐implantation risk factors that predict mortality. However, most studies have focused on isolated risk factors and their effect on mortality.^4^, ^5^, ^6^ The VALID‐CRT risk score, which is based on routine, readily available, clinical variables, has proved to reliably predict long‐term total and cardiovascular mortality in patients undergoing CRT.^7^ The aim of the present study was to confirm in an unselected real‐world CRT population, the value of the VALID‐CRT risk score in predicting outcome, and to assess its association with clinical and echocardiographic response, to identify those patients who achieved a satisfactory “reverse remodeling” after CRT.

## 2 METHODS

The CRT MOdular Registry (CRT MORE) (ClinicalTrials.gov Identifier: NCT01573091) was a prospective, single‐arm, multi‐center cohort study designed to evaluate the association between baseline and implantation variables and the outcomes of patients in whom a CRT device has been implanted in accordance with current guidelines at the time of implantation.^8^ Enrollment started in December 2011 and ended in November 2013. The design of the study has been published previously.^9^

### 2.1 Risk‐stratification algorithm

The VALID‐CRT risk score 7 was constructed and validated on the following variables: age, gender, ICD back‐up, atrial fibrillation, presence, or absence of atrioventricular junction ablation in the case of atrial fibrillation, ischemic etiology, diabetes, New York Heart Association (NYHA) class, and left ventricular ejection fraction (LVEF). For analysis purpose, according with the VALID‐CRT predictor index (PI) based on the quintiles of the patient's distribution, five groups of increasing risks were chosen to balance interest with adequate sample sizes. First, we applied the VALID‐CRT risk score cutoff to create five groups (quintiles Q1‐Q5) according to the centile (≤20th; 21st‐40th; 41st‐60th; 61st‐80th; ≥81st) of the PI. We then applied the VALID‐CRT risk stratification model to CRT‐MORE patients, calculating a specific PI for our study population, and creating five risk groups according to the centile (quintiles Q1‐Q5). We used this specific CRT‐MORE population‐based PI to evaluate the ability to predict outcome and CRT response in the CRT‐MORE population.

### 2.2 End‐points

The primary end‐point of the study was all‐cause death. Secondary end‐points were: (a) death from any cause and HF hospitalization, whichever occurred first after CRT implantation; (b) HF hospitalization; (c) clinical response at 12‐month follow‐up; (d) echocardiographic evaluation at 12‐month follow‐up.

In detail, the clinical response was assessed in accordance with a hierarchical composite criterion comprising live status, hospitalization for HF, and variations in NYHA functional class. Specifically, a positive response was attributed to patients who remained alive without any episode of HF hospitalization after 12 months of CRT delivery and showed an improvement in NYHA class or remained in NYHA class I or II. Patients who died or were hospitalized for signs of HF, showed worsening of their NYHA class or remained in NYHA class III or IV were classified as non‐responders. LV reverse remodeling was evaluated by measuring the effect of CRT on LV end‐systolic volume (LVESV) and on LVEF, by comparing the baseline value with that recorded at the 12‐month follow‐up examination of surviving patients, and by calculating the proportion of patients who displayed a relative reduction of 15% or more in LVESV and an absolute increase in LVEF of more than 5%.

### 2.3 Statistical analysis

Descriptive statistics are reported as mean ± SD for normally distributed continuous variables, or medians with 25th to 75th percentiles in the case of skewed distribution. Normality of distribution was tested by means of the nonparametric Kolmogorov‐Smirnov test. Differences between mean data were compared by means of a *t* test for Gaussian variables, using the *F*‐test to check the hypothesis of equality of variance. The Mann‐Whitney non‐parametric test was used to compare non‐Gaussian variables. Differences in proportions were compared by applying χ^2^ analysis or Fisher's exact test, as appropriate. Odds ratios (ORs) with corresponding 95% confidence intervals (CIs) are reported. The cumulative probability of HF or death was displayed by the method of Kaplan‐Meier, and the log‐rank test was used to compare cumulative events. Hazard ratios (HRs) and their 95% CIs were computed by means of a Cox regression model, in which risk‐group variables were fixed covariates and deaths or cardiovascular hospitalizations were time‐dependent covariates. A two‐sided *P*‐value <.05 was considered statistically significant for single tests. All statistical analyses were performed by means of STATISTICA software, version 7.1 (StatSoft, Inc., Tulsa, OK).

## 3 RESULTS

### 3.1 Study population

The clinical characteristics of the study population are summarized in Table 1. The median age of the 905 patients enrolled was 72 (64‐77) years; 667/905 (73.7%) were male, and 548 (60.6%) were in NYHA class III/IV.

**Table 1:** Demographics and baseline characteristics of the study population

Parameter	n = 905
Age, years	72 (64‐77)
Males, n (%)	667 (73.7)
BMI	26.5 (24‐29)
NYHA III/IV, n (%)	548 (60.6)
Ischemic, n (%)	424 (46.9)
Dilated, n (%)	403 (44.5)
Other, n (%)	78 (8.6)
COPD n (%)	215 (23.8)
CKD, n (%)	235 (26)
eGFR (mL/min)	62 (43‐85)
Diabetes, n (%)	278 (30.7)
Hypertension, n (%)	593 (65.5)
AF history, n (%)	190 (21)
Permanent AF, n (%)	126 (14)
HR, bpm	68 (60‐76)
QRS, ms	158 (10‐170)
PR, ms	180 (160‐203)
LBBB, n (%)	749 (82.8)
Statins, n (%)	412 (45.5)
Betablockers, n (%)	722 (79.8)
ACE‐ARB, n (%)	676 (74.7)
Loop diuretics, n (%)	677 (74.8)
aldosterone receptor antagonists (ARA), n (%)	417 (46.1)
Antiarrhythmics, n (%)	212 (23.4)
Ivabradine, n (%)	66 (7.3)
LVEDD, mm	63 (59‐69)
LVESD, mm	52 (46‐58)
LVEDV, mL	176 (140‐225)
LVESV, mL	125 (95‐164)
LVEF	30 (25‐34)
mitral regurgitation (MR) grade ≥ 2, n (%)	442 (48.8)
left atrial diameter (LAD), mm	46 (41‐52)
CRT‐D, n (%)	798 (88.2)
RV apex, n (%)	597 (66)
LV lateral, n (%)	485 (53.6)
LV anterior, n (%)	194 (21.4)
LV posterior, n (%)	226 (25)

Abbreviations: ACE‐ARB, angiotensin converting enzyme‐angiotensin‐receptor blockers; AF, atrial fibrillation; BMI, body mass index; CKD, chronic kidney disease; COPD, chronic obstructive pulmonary disease; CRT, cardiac resynchronization therapy; eGFR, estimated glomerular filtration rate; HR, hazard ratio; LV, left ventricular; LVEDD, left ventricular end‐diastolic diameter; LVEDV, left ventricular end‐diastolic volume; LVEF, left ventricular ejection fraction; LVESD, left ventricular end‐systolic diameter; LVESV, left ventricular endsystolic volume; NYHA, New York Heart Association; RV, right ventricular.

### 3.2 Follow‐up

The median follow‐up duration was 1005 [627‐1361] days. By the end of the study, 134 (14.8%) patients had died and 79 (8.7%) had been hospitalized for HF. The combined end‐point of death or HF hospitalization was reached by 199 (22%) patients. Clinical response data were available for 836 (92.4%) patients. Of these, 579 (69.3%) patients displayed an improvement in their clinical status at 12‐month follow‐up, and were classified as responders to CRT. Data on LVEF evaluation were available for 737 patients (81.4%), while data on LVESV evaluation were available for 569 patients (62.9%). Of these, 417 (56.6%) and 344 (60.5%) were classified as responders with regard to LVEF and LVESV endpoints, respectively.

### 3.3 Risk stratification according to VALID‐CRT PI cutoff points

**Figure 1:**
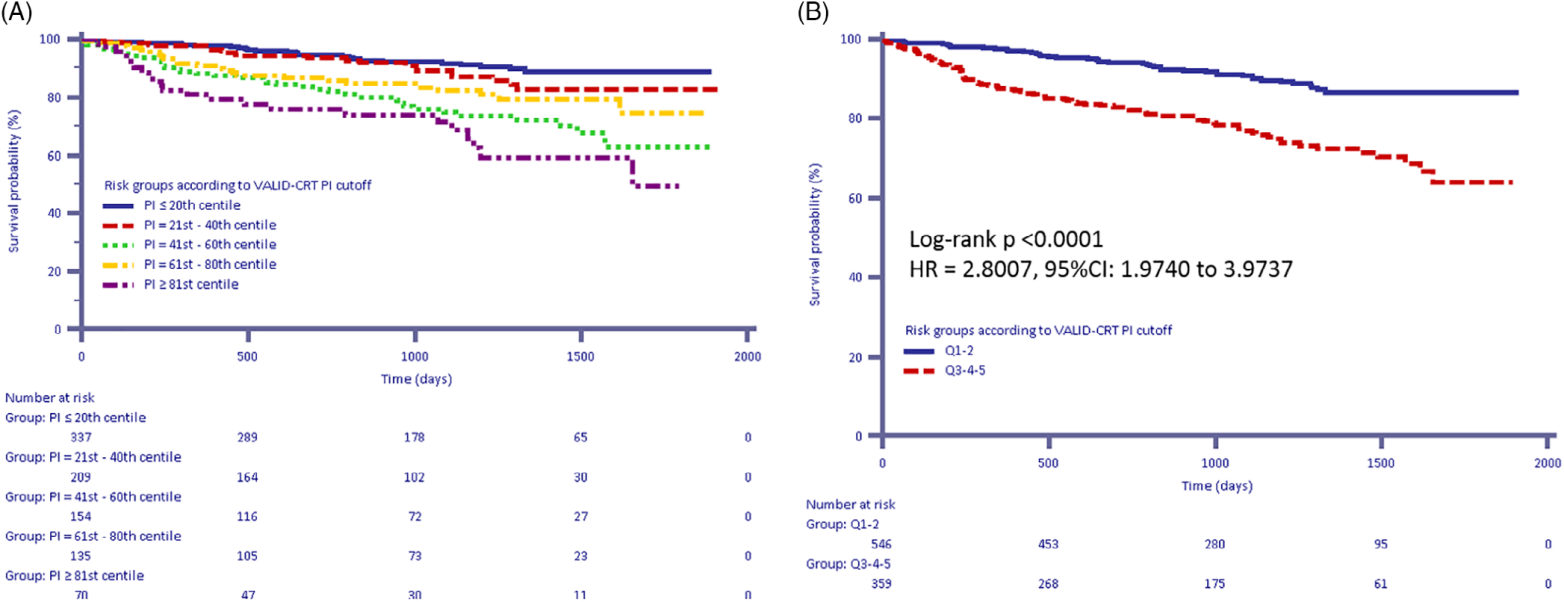
A, B Kaplan‐Meier estimates of time to death from any cause according to VALID‐cardiac resynchronization therapy predictor index cutoff values. A, Quintiles 1‐5; B: Quintiles 1‐2 vs Quintiles 3‐5

Risk stratification of the CRT‐MORE population according to VALID‐CRT PI cutoff points is reported in Table S1, while Kaplan‐Meier survival curves of the CRT‐MORE population according to VALID‐CRT PI cutoff points is depicted in Figure 1. Although it had been derived in a population with different clinical characteristics, the VALID‐CRT Risk Score well stratified the outcome of patients at low risk (Q1 and Q2) in comparison with those at intermediate‐to‐high risk (Q3‐Q5) (log‐rank *P* < .0001; HR Q3‐5 vs Q1‐2:2.8; 95% CI 1.97‐3.97). Both the percentage of patients who died from any cause during the 2‐year follow‐up period (5.3% vs 16.3%, *P* < .0001) and total mortality (9% vs 23.7%, *P* < .0001) were significantly different between low‐risk and intermediate/high‐risk.

### 3.4 Risk‐stratification algorithm according to CRT‐MORE population‐based PI cutoff points

**Figure 2:**
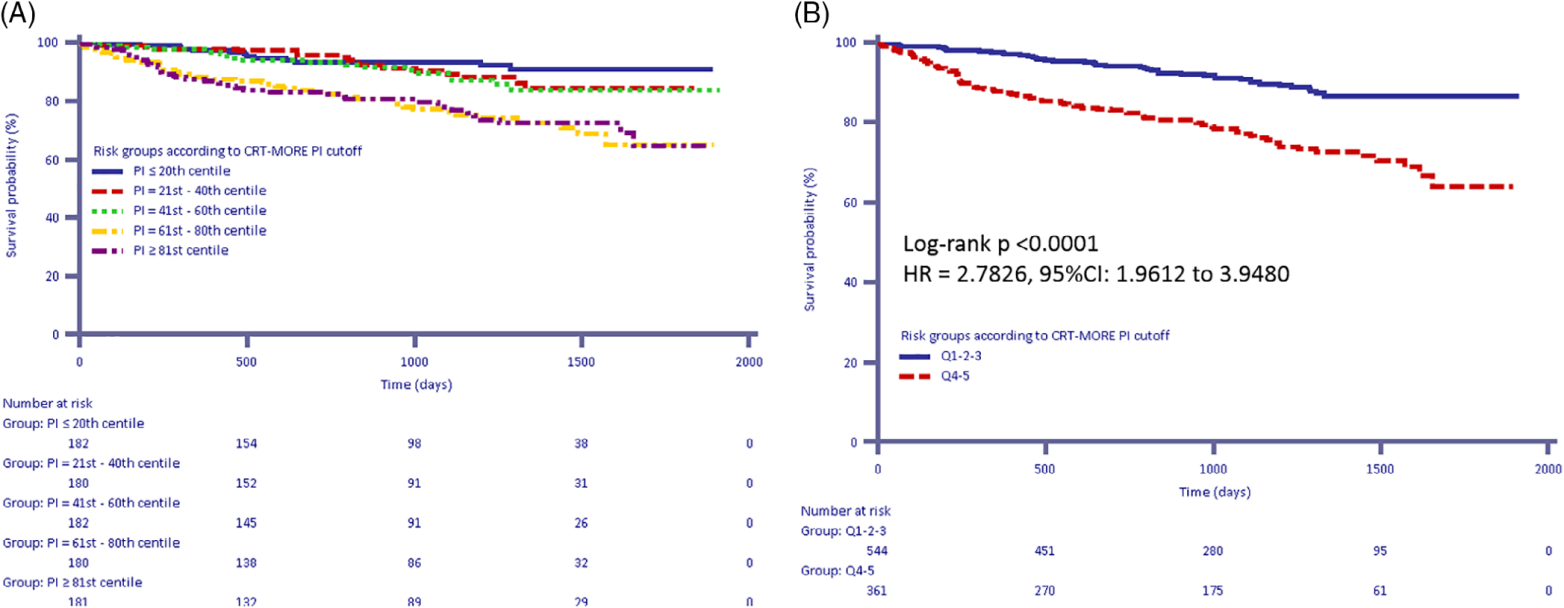
A, B, Kaplan‐Meier estimates of time to death from any cause according to the cardiac resynchronization therapy MOdular Registry population‐based predictor index . A, Quintiles 1‐5; B, Quintiles 1‐3 vs Quintiles 4‐5

**Figure 3:**
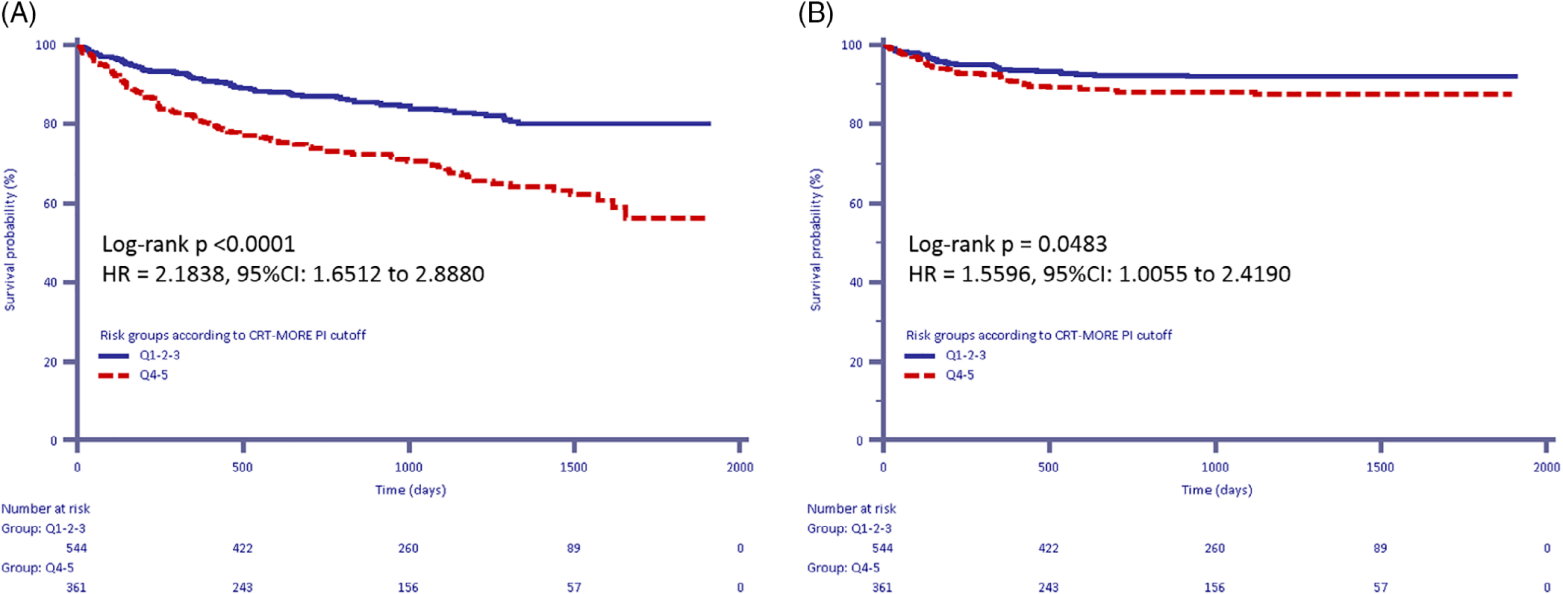
A, B, Kaplan‐Meier estimates of time to the combined endpoint of death from any cause and HF hospitalizations according to the cardiac resynchronization therapy MOdular Registry (CRT‐MORE) population‐based predictor index (PI) (A) and Kaplan‐Meier estimates of time to heart failure hospitalizations according to the CRT‐MORE population‐based PI (B)

**Figure 4:**
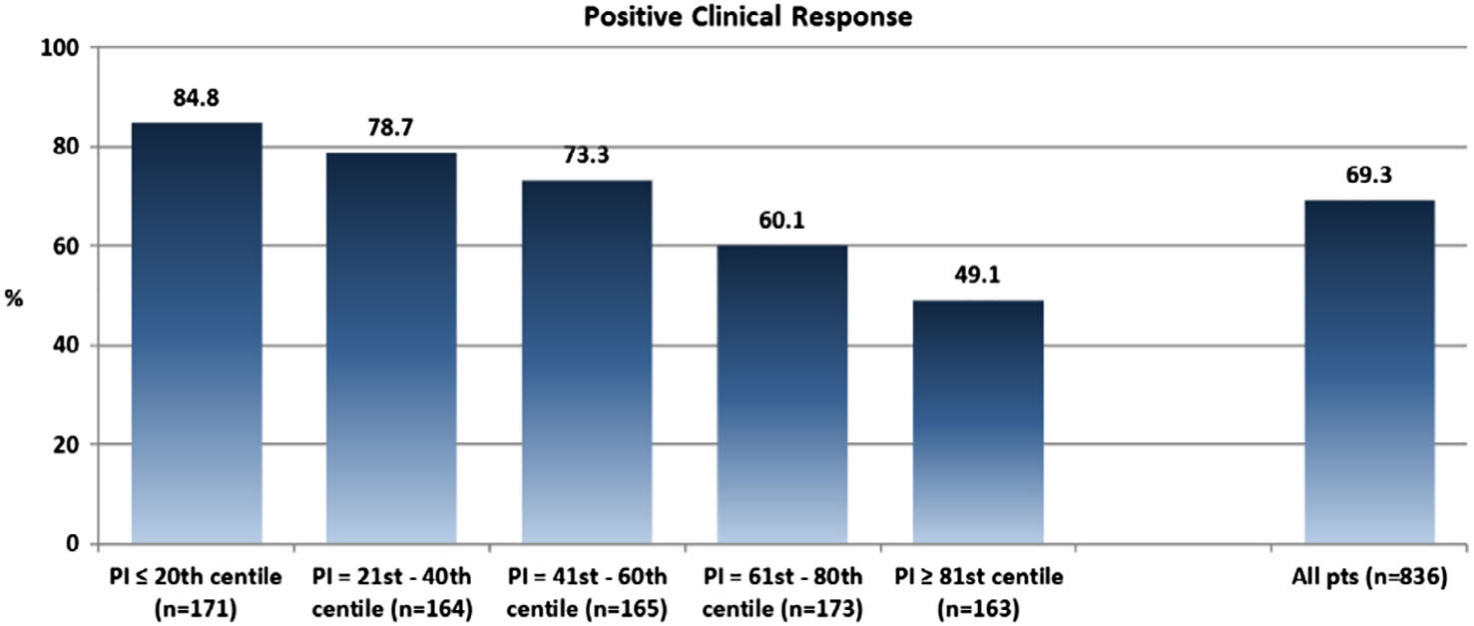
Clinical Response at 12‐month follow‐up according to the severity of the cardiac resynchronization therapy MOdular Registry population‐based predictor index

On applying the risk‐stratification model to the CRT‐MORE population, a median PI of 0.353 [−0.247‐0.870] was found. The ability of the risk‐stratification algorithm to predict the study end‐points is displayed in Table S2. On plotting the mean survival according to quintiles of the PI, a clear separation of curves emerged between the three highest quintiles and the two lowest quintiles (Figure 2A, B) (log‐rank *P* < .0001; HR Q1‐3 vs Q4‐5:2.78; 95% CI 1.96‐3.95). The same picture was reproduced on plotting survival free from death from any cause and hospitalization for HF (Figure 3A) (log‐rank *P* < .0001; HR Q1‐3 vs Q4‐5:2.18; 95% CI 1.65‐2.89), while on plotting only survival free from hospitalization for HF, the separation among the curves was less marked (Figure 3B), (*P* = .0483; HR Q1‐3 vs Q4‐5:1.56; 95% CI 1.01‐2.42). The clinical response was significantly lower in pts with high‐to‐very high PI (Q4‐5) than in pts with low‐to‐intermediate PI (Q1‐3) (55% vs 79%, *P* < .0001), and declined as the PI increased (ranging from 85%‐Q1‐ to 49%‐Q5, Figure 4). By contrast, we did not find any association between PI and echocardiographic response (positive LVEF remodeling ranging from 53.5%‐Q1 to 59.4%‐Q5, positive LVESV remodeling ranging from 60.9%‐Q1 to 55.6%‐Q5, both with variable trend among quintiles, *P* = NS; Figure S5).

## 4 DISCUSSION

### 4.1 Main findings

In a real‐world cohort of HF patients in whom a CRT device had been implanted, we found that the VALID‐CRT risk score predicted not only total and cardiovascular mortality, but also clinical response to CRT.

### 4.2 Value of risk‐stratification algorithms in predicting total and cardiovascular mortality after CRT

While several risk‐stratification algorithms have been validated since CRT became a usual therapy for HF patients, the VALID‐CRT risk score has proved to be the most reliable. Seven indeed, in an external validation of a large cohort of CRT patients on long‐term follow‐up, it discriminated well between risk groups (*c*‐statistic 0.70). Seven another score, the DSC index, which combined pre‐implant measures of dyssynchrony and location of myocardial scar as well as creatinine, demonstrated to be a novel, powerful predictor of cardiovascular mortality.^10^ The SHFM, a multifactorial model that predicts mortality in patients with HF,^11^ performed modestly when subjected to external multicenter validation in patients undergoing CRT, tending to overestimate survival.^12^ Three other simple scores proved to readily predict survival after CRT. The EAARN score, a simple score based on LVEF, age, atrial fibrillation, renal dysfunction, and NYHA class IV had a significant add‐on predictive effect with regard to mortality, but has not been externally validated.^13^ The HF‐CRT score, which combined clinical and readily available biomarker data, stratified CRT patients for HF progression and death.^14^ The CRT‐SCORE, based on clinical, electrocardiographic, and echocardiographic parameters, accurately predicted 1‐ and 5‐year survival rates after CRT.^15^

Although our population comprised a lower percentage of patients in NYHA class III‐IV (60.6% vs 77.5%) than the population used to derive and validate the VALID‐CRT risk score, our application of this score revealed a clear separation of curves of survival from death and from the combination of death and hospitalization for HF between the three highest quintiles and the two lowest quintiles (Figures 2 and 3).

### 4.3 Value of risk‐stratification algorithms in predicting clinical and echocardiographic response after CRT

While almost all previously published risk scores predict survival after CRT, none of them has so far been able to identify those patients who are most likely to respond to CRT. On applying the VALID‐CRT risk score, we were able to select patients with a high‐to‐very high‐risk profile, only 55% of whom had improved 12 months after implantation. These patients should not be excluded from receiving CRT therapy, but might require more intensive follow‐up and more aggressive therapy than those with a low‐risk profile, and probably should receive only a CRT‐P for the high risk of death for HF.

We did not find any association between PI and echocardiographic response, in terms of either LVEF remodeling or LVESV remodeling. The ability of a clinical and echocardiographic score to predict left ventricular remodeling has never been assessed. Very recently, in a cohort of 491 patients, Vegh et al found that a score based on the pre‐ and post‐implantation 12‐lead surface ECG had an independent value in predicting reverse remodeling of the left ventricle and long‐term survival free from HF hospitalization or transplantation.^16^ This ECG score is based on QRS duration shortening, intrinsic deflection time and post‐pacing change in R + S amplitude. The score is applicable regardless of the intrinsic conduction block pattern on baseline ECG, but seems to be more robust in patients with an a priori left bundle branch block (LBBB).

### 4.4 Study limitations

The study has several potential limitations. First, as patients were followed up over an average of 1005 days, further studies will be needed to validate the longer‐term consistency of the present results in terms of benefit from CRT therapy. Second, the echocardiographic response to CRT was not evaluated in a core laboratory. Nevertheless, the baseline and follow‐up echocardiographic evaluations were performed by the same operator in each patient. Third, data on LVESV evaluation were available only for 62.9% of our population: this limitation could have prevented VALID CRT score to identify those patients who obtained reverse remodeling.

## 5 CONCLUSIONS

The VALID‐CRT risk‐stratification algorithm reliably predicts outcome and CRT response after CRT in an unselected, real‐world population and it may be useful in tailoring follow‐up and treatment strategies in clinical practice. In particular, high‐to‐very high‐risk profile patients should undergo intensive follow‐up in order to improve their clinical response.

## CONFLICT OF INTEREST

Maurizio Malacrida is an employee of Boston Scientific. No other conflicts of interest exist.

## Supporting information


**TABLE S1** Risk‐stratification groups according to VALID‐CRT PI cut‐off points.

**TABLE S2**. Predictive value of risk‐stratification algorithm according to CRT‐MORE population‐based PI cutoff points for the study end‐points

**FIGURE S5 A, B** Echocardiographic Response at 12‐month follow‐up according to the severity of the CRT‐MORE population‐based PI. A, LVESV remodeling. B, LVEF remodeling.
