# Supplementary material for: The VALID‐CRT risk score reliably predicts response and outcome of cardiac resynchronization therapy in a real‐world population
Source: Clin Cardiol. 2019 Jul 13;42(10):919–24. doi: 10.1002/clc.23229 (PMC6788573; doi:10.1002/clc.23229)
Supplement: Supplementary file 1 — TABLE S1 Risk‐stratification groups according to VALID‐CRT PI cut‐off points. [file CLC-42-919-s001.docx]

### Supplementary Table 1. Risk-stratification groups according to VALID-CRT PI cut-off points.

|  | % Pts | Total mortality (%) | 2-year mortality (%) | Mortality Annual rate (100 person-years) |
| --- | --- | --- | --- | --- |
| Risk group 1 (n=337) | 37.2 | 8.0 | 5.0 | 2.8 |
| Risk group 2 (n=209) | 23.1 | 10.5 | 5.7 | 4.1 |
| Risk group 3 (n=154) | 17.0 | 24.7 | 16.2 | 9.6 |
| Risk group 4 (n=135) | 14.9 | 17.8 | 12.6 | 6.6 |
| Risk group 5 (n=70) | 7.7 | 32.9 | 22.9 | 14.2 |
|  |  |  |  |  |
| All pts (n=905) | 100.0 | 14.8 | 9.6 | 5.6 |
