# Supplementary material for: The VALID‐CRT risk score reliably predicts response and outcome of cardiac resynchronization therapy in a real‐world population
Source: Clin Cardiol. 2019 Jul 13;42(10):919–24. doi: 10.1002/clc.23229 (PMC6788573; doi:10.1002/clc.23229)
Supplement: Supplementary file 2 — TABLE S2. Predictive value of risk‐stratification algorithm according to CRT‐MORE population‐based PI cutoff points for the study end‐points [file CLC-42-919-s002.docx]

### Supplementary Material: Table 2. Predictive value of risk-stratification algorithm according to CRT-MORE population-based PI cut-off points for the study end-points

| **Endpoint** | **HR** | **95%CI** | ***p* value** |
| --- | --- | --- | --- |
| Death from any cause  HF hospitalizations  Combined endpoint | 2.0262  1.3841  1.7282 | 1.6163 to 2.5402  1.0427 to 1.8372  1.4431 to 2.0697 | < 0.0001  0.0252  < 0.0001 |
| **Endpoint** | **OR** | **95%CI** | ***p* value** |
| Clinical response at 12 months | 0.4128 | 0.3329 to 0.5119 | < 0.0001 |
| LVEF improvement at 12 months | 1.1198 | 0.9247 to 1.3561 | 0.2466 |
| LVESV improvement at 12 months | 0.9777 | 0.7787 to 1.2275 | 0.8461 |
