# Supplementary figures and images for: The VALID‐CRT risk score reliably predicts response and outcome of cardiac resynchronization therapy in a real‐world population
Source: Clin Cardiol. 2019 Jul 13;42(10):919–24. doi: 10.1002/clc.23229 (PMC6788573; doi:10.1002/clc.23229)

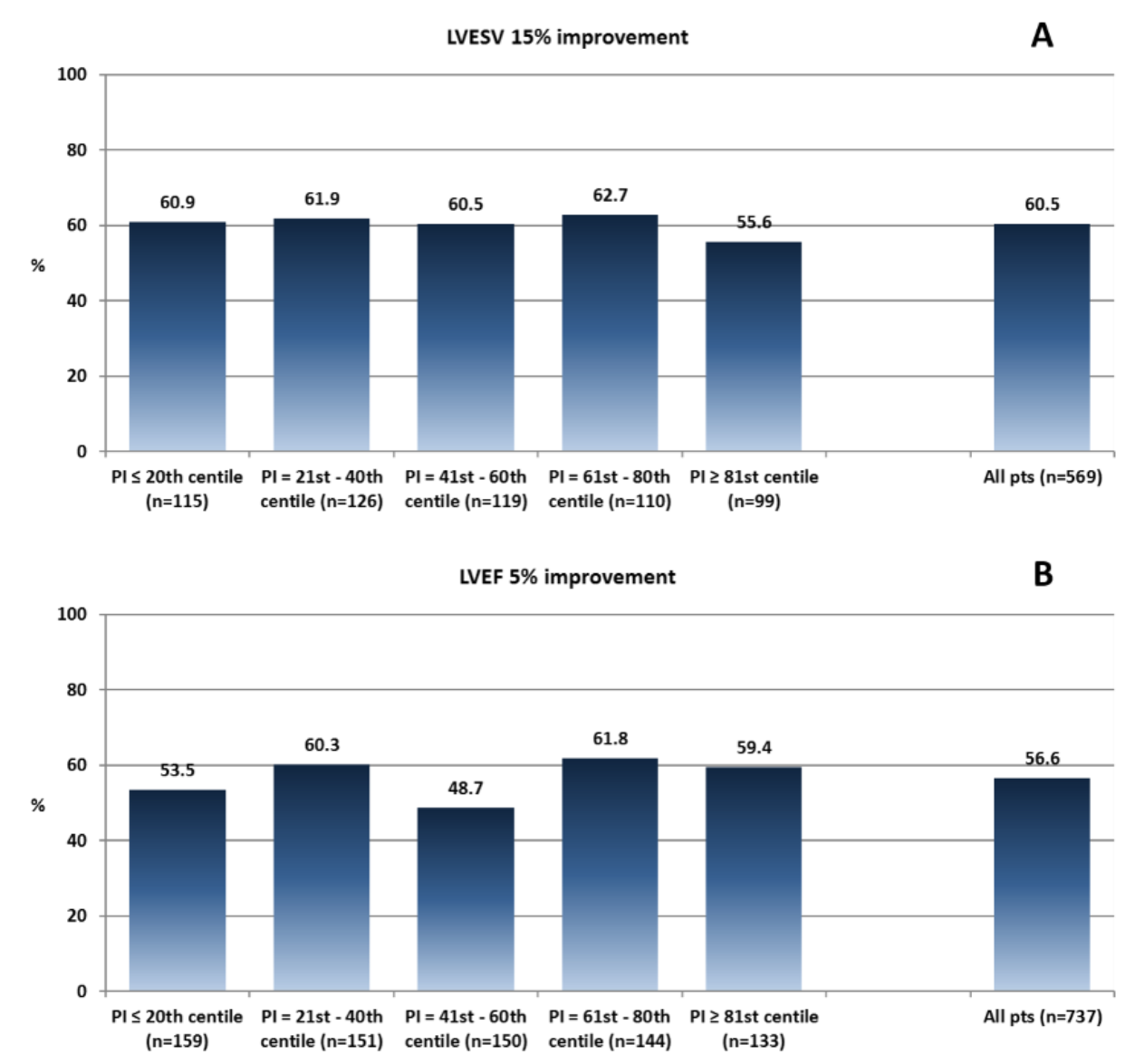

Supplement: Supplementary file 3 — FIGURE S5 A, B Echocardiographic Response at 12‐month follow‐up according to the severity of the CRT‐MORE population‐based PI. A, LVESV remodeling. B, LVEF remodeling. [file CLC-42-919-s003.tif]
